# BayCANN: Streamlining Bayesian Calibration With Artificial Neural Network Metamodeling

**DOI:** 10.3389/fphys.2021.662314

**Published:** 2021-05-25

**Authors:** Hawre Jalal, Thomas A. Trikalinos, Fernando Alarid-Escudero

**Affiliations:** ^1^Department of Health Policy and Management, University of Pittsburgh, Graduate School of Public Health, Pittsburgh, PA, United States; ^2^Departments of Health Services, Policy & Practice and Biostatistics, Brown University, Providence, RI, United States; ^3^Division of Public Administration, Center for Research and Teaching in Economics (CIDE), Aguascalientes, Mexico

**Keywords:** Bayesian calibration, machine learning, mechanistic models, artificial neural networks, emulators, surrogate models, metamodels

## Abstract

**Purpose:** Bayesian calibration is generally superior to standard direct-search algorithms in that it estimates the full joint posterior distribution of the calibrated parameters. However, there are many barriers to using Bayesian calibration in health decision sciences stemming from the need to program complex models in probabilistic programming languages and the associated computational burden of applying Bayesian calibration. In this paper, we propose to use artificial neural networks (ANN) as one practical solution to these challenges.

**Methods:** Bayesian Calibration using Artificial Neural Networks (BayCANN) involves (1) training an ANN metamodel on a sample of model inputs and outputs, and (2) then calibrating the trained ANN metamodel instead of the full model in a probabilistic programming language to obtain the posterior joint distribution of the calibrated parameters. We illustrate BayCANN using a colorectal cancer natural history model. We conduct a confirmatory simulation analysis by first obtaining parameter estimates from the literature and then using them to generate adenoma prevalence and cancer incidence targets. We compare the performance of BayCANN in recovering these “true” parameter values against performing a Bayesian calibration directly on the simulation model using an incremental mixture importance sampling (IMIS) algorithm.

**Results:** We were able to apply BayCANN using only a dataset of the model inputs and outputs and minor modification of BayCANN's code. In this example, BayCANN was slightly more accurate in recovering the true posterior parameter estimates compared to IMIS. Obtaining the dataset of samples, and running BayCANN took 15 min compared to the IMIS which took 80 min. In applications involving computationally more expensive simulations (e.g., microsimulations), BayCANN may offer higher relative speed gains.

**Conclusions:** BayCANN only uses a dataset of model inputs and outputs to obtain the calibrated joint parameter distributions. Thus, it can be adapted to models of various levels of complexity with minor or no change to its structure. In addition, BayCANN's efficiency can be especially useful in computationally expensive models. To facilitate BayCANN's wider adoption, we provide BayCANN's open-source implementation in R and Stan.

## 1. Background

Modelers and decision-makers often use mathematical simulation models to simplify real-life complexity and inform decisions, particularly those for which uncertainty is inherent. However, some of the model parameters might be either unobserved or unobservable due to various financial, practical or ethical reasons. For example, a model that simulates the natural history of cancer progression may lack an estimate for the rate at which an individual transitions from a pre-symptomatic cancer state to becoming symptomatic. Although this rate might not be directly observable, it may be estimated using a technique commonly referred to as calibration (Alarid-Escudero et al., 2018; Vanni et al., 2011; Rutter et al., 2009). Thus, calibration involves modifying the model input parameters until the desired output is obtained.

Calibration has the potential for improving model inference, and recent guidelines recommend that model calibration of unknown parameters should be performed where data on outputs exist (Weinstein et al., 2003; Briggs et al., 2012). Modelers are also encouraged to report the uncertainty around calibrated parameters and use these uncertainties in both deterministic and probabilistic sensitivity analyses (Briggs et al., 2012).

There are several calibration techniques with various levels of complexity. For example, Nelder-Mead is a direct-search algorithm commonly used to calibrate models in health and medicine. Nelder-Mead is a deterministic approach that searches the parameter space for good-fitting parameter values (Nelder and Mead, 1965). Although Nelder-Mead is generally effective, it cannot estimate parameter distributions or directly inform on the correlations among the calibrated parameters. It is also not guaranteed to find a global optimal value because it might converge to a local optimum. Unlike the direct-search algorithms, Bayesian methods are naturally suited for calibration because they estimate the input parameter's posterior joint and marginal distributions (Menzies et al., 2017). However, Bayesian methods are difficult to implement due to the complexity of the models used and the computational challenges of applying these methods. Bayesian calibration often requires tens or hundreds of thousands of simulation runs and a model written in a probabilistic programming language, such as Stan (Carpenter et al., 2017) or Bayesian inference Using Gibbs Sampling (BUGS) (Lunn et al., 2009). We argue that the complexity of these tasks and their potential computational demand have prevented a wider adoption of Bayesian calibration methods in health decision science models.

In this manuscript, we use artificial neural network (ANN) metamodels as a practical approach to streamlining Bayesian calibration in complex simulation models. Metamodels have increasingly been used to overcome the computational burden of Bayesian calibration. A metamodel is a surrogate model that can be used to approximate the model's input-output relationship (Jalal et al., 2013). Metamodels can provide an approximation to the simulation model in a fraction of the time. While ANN metamodels are not fully probabilistic, they are flexible functions that can map highly non-linear relationships in large data. We use an ANN metamodel as an emulator to substitute the simulation model in the Bayesian calibration analysis. Thus, the ANN acts as a low computational cost proxy of the simulation model. In addition, analysts do not need to program their simulation model in a probabilistic language because coding the ANN in probabilistic languages (e.g., Stan) is relatively straight-forward, and analysts can reuse the provided Stan code with little or no modification.

We refer to our approach as Bayesian calibration via artificial neural networks, or BayCANN for short. We demonstrate BayCANN by calibrating a realistic model of the natural history of colorectal cancer (CRC). We compare this approach's results to an approximate Bayesian calibration of the original model using an incremental mixture importance sampling (IMIS) algorithm. We provide the code in R and Stan for our application that researchers can adapt to calibrate their models.

## 2. Methods

We start this exposition by reviewing elements of Bayesian calibration. We describe the computational burden of using Bayes theorem in most realistic models, and how deep ANNs can streamline Bayesian calibration methods to calibrate these models. We illustrate this approach by calibrating a natural history model of CRC. We also compare BayCANN's performance to a Bayesian calibration using IMIS directly on a simulation model.

### 2.1. Bayesian Calibration

The Bayes theorem states that

(1) $$\begin{array}{r} p\left(\theta |\text{data}\right)=\frac{p\left(\text{data}|\theta \right)p\left(\theta \right)}{p\left(data\right)}, \end{array}$$

where θ is a set of model parameters, data is the observed data, and *p*(data|θ) is the same as the likelihood *l*(θ|data). Because the denominator is not a function of θ, we can rewrite Equation (1) as

(2) $$\begin{array}{r} p\left(\theta |\text{data}\right)\propto l\left(\theta |\text{data}\right)p\left(\theta \right). \end{array}$$

Table 1 shows how each term in Equation (2) can be mapped to a component in a calibration exercise. The prior distribution, *p*(θ), represents our uncertainty about the distribution of the model parameters before calibrating the model. Modelers often use various distributions to describe this uncertainty, including beta or logit-normal distribution for probabilities, gamma for rates, or log-normal distributions for rates or hazard ratios. Thus, we can think of a prior distribution as the uncertainty of the pre-calibrated model input parameters. For example, we can represent a vague distribution by a uniform distribution where all the values are equally likely within a defined range.

**Table 1:** The Bayes formula in a calibration context.

**Term**	**Bayesian context**	**Calibration context**
*p*(θ)	Prior distribution of the model input parameters θ	Pre-calibrated model input parameters
*p*(θ|data)	Posterior distribution of the model parameters θ given observed data	Calibrated model parameters to target data
*l*(θ|data)	Probability of the data given model parameters θ (model likelihood)	Objective function or goodness-of-fit measure; how well the model output fits the target data given a particular value of θ

Bayesian calibration will update the prior distribution based on the observed target data. The term *p*(θ|*data*) is called the posterior distribution, representing the updated distribution of θ after observing some data. The posterior distribution is equivalent to the calibrated parameter distribution when the data are the calibration targets.

The likelihood function, *l*(θ|*data*), denotes how likely the observed data arise from a given data generation mechanism with a parameter set values θ. From a simulation modeling perspective, *l*(θ|*data*) is equivalent to measuring the goodness of the model output fit to the calibration targets given a simulation model's input parameter set θ.

Thus, we can map all components of Bayes theorem to calibration components and use Bayesian inference to obtain the calibrated parameter distributions (a.k.a. the posterior distributions).

Bayesian calibration is often challenging to adopt in practice in health decision science models. The main challenge lies in the complexity of applying Equation (2). Specifically, an analytical solution for *p*(θ|data) is unlikely to exist for most realistic simulation models. Thus, specialized algorithms, such as Markov-Chain Monte-Carlo (MCMC) might be necessary at the expense of being practically challenging to implement for complex models and computationally expensive.

### 2.2. Metamodels

To overcome the computational and practical challenges of Bayesian calibration, we propose to use artificial neural network (ANN) metamodels. As described above, a metamodel is a surrogate model that approximates the relationship between the simulation model's inputs and outputs (i.e., a metamodel is a model of the model) (Blanning, 1974; Kleijnen, 1975; Kleijnen et al., 2005; Kleijnen, 2015). Metamodels range from simple models, such as linear regressions, to complex non-linear models, such as artificial neural networks (ANN). Although linear regression models are the most common form of metamodels (Barton and Meckesheimer, 2006; Barton, 2009; Sacks et al., 1989; Fu, 1994; Weiser Friedman, 1996; Banks, 1998; Kleijnen and Sargent, 2000; Jalal et al., 2013, 2015), in this paper we focus on ANN because they are more flexible while still being relatively simple to implement in Stan or BUGS.

Metamodels are often used because they generally offer a vast reduction in computation time (Kleijnen, 1979; Friedman and Pressman, 1988; Barton, 1992; Weiser Friedman, 1996; O'Hagan et al., 1999; Barton and Meckesheimer, 2006; Santos and Santos, 2007; Reis dos Santos and Reis dos Santos, 2009; Khuri and Mukhopadhyay, 2010). For example, a model that takes several hours or even days to run can be approximated with a metamodel that may only take a few milliseconds. This feature has been an attractive attribute of metamodels for many decades in engineering and computer science. Examples of metamodels in health decision sciences involve revealing model uncertainty using linear regression mdetamodeling (Jalal et al., 2013), and speeding up computationally expensive microsimulation models using Gaussian processes metamodeling (Stevenson et al., 2004; de Carvalho et al., 2019).

An additional benefit of using metamodels for Bayesian calibration is that one can reuse the same metamodel structure to calibrate very different simulation models. The same BayCANN code can be adapted to other problems with no or minimal change.

#### 2.2.1. ANN Metamodels

Artificial neural networks (ANNs) are networks of non-linear regressions that were initially developed to mimic the neural signal processing in the brain and to model how the nervous system processes complex information (Másson and Wang, 1990; Michie et al., 1994; Rojas, 1996; Jain et al., 1996; Olden et al., 2008). ANNs have recently witnessed significant advances for applications in machine learning, artificial intelligence, and pattern recognition (Ravì et al., 2016).

**Figure 1:**
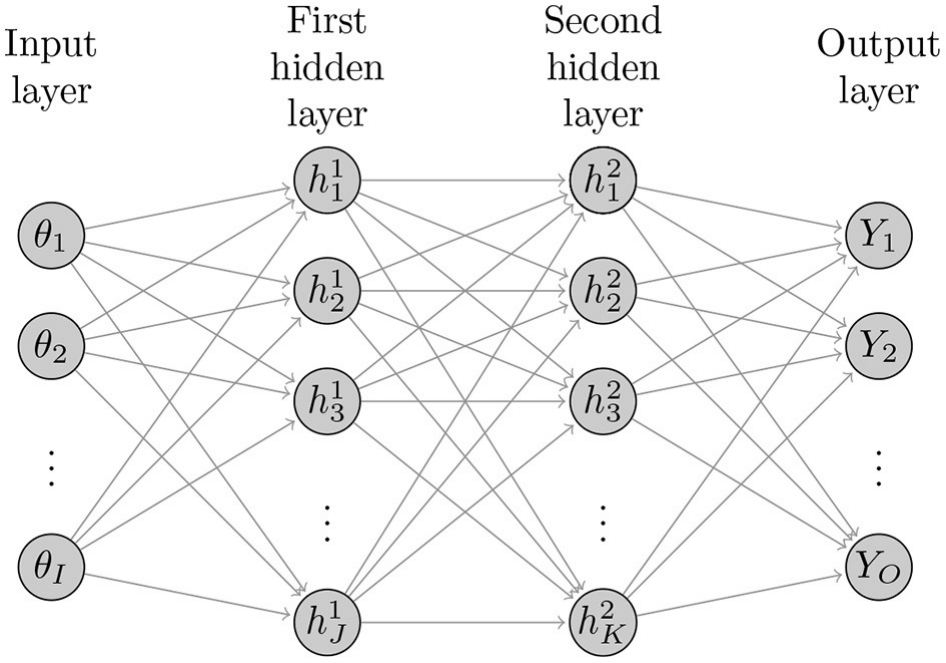
Diagram of general structure of a deep neural network with *I* inputs, two hidden layers with *J* and *K* hidden nodes and *O* outputs.

Figure 1 illustrates the basic structure of a four-layer neural network with two hidden layers with *I* neurons (nodes) in the input layer, *J* hidden nodes in the first hidden layer, *K* hidden nodes in the second hidden layer, and *O* output nodes in the output layer. The ANNs with more than one hidden layer are often referred to as deep ANNs. The following sets of equations represent the structure of this ANN

(3) $$\begin{array}{l} \begin{array}{l} z^{\left(1\right)}=W^{\left(1\right)}\theta +b^{\left(1\right)}\\ h^{\left(1\right)}=f^{\left(1\right)}\left(z^{\left(1\right)}\right)\\ z^{\left(2\right)}=W^{\left(2\right)}h^{\left(1\right)}+b^{\left(2\right)}\\ h^{\left(2\right)}=f^{\left(2\right)}\left(z^{\left(2\right)}\right)\\ z^{\left(3\right)}=W^{\left(3\right)}h^{\left(2\right)}+b^{\left(3\right)}\\ \;Y=f^{\left(3\right)}\left(z^{\left(3\right)}\right), \end{array} \end{array}$$

where θ is the simulation model inputs, *Y* is the model outputs to be compared to the calibrated targets, and (*W, b*) = (*W*^(1)^, *b*^(1)^, *W*^(2)^, *b*^(2)^, *W*^(3)^, *b*^(3)^) are the ANN coefficients. *W*^(1)^ are the weights connecting the inputs θ with the nodes in the first hidden layer, *W*^(2)^ are the weights connecting the nodes in the first and second hidden layers, and *W*^(3)^ are the weights connecting the nodes in the second hidden layer with the output *Y*. The terms *b*^(1)^, *b*^(2)^ and *b*^(3)^ are corresponding bias (intercept) terms. *f*^(1)^ is the activation function, commonly, a sigmoid function such as a hyperbolic tangent function

(4) $$\begin{array}{r} f^{\left(1\right)}\left(z^{\left(1\right)}\right)=\frac{2}{1+e^{-2z^{\left(1\right)}}}-1. \end{array}$$

The function *f*^(3)^ is called a transfer function that transforms the results from the last hidden layer's nodes into a working output. The transfer function can also be a sigmoid function or a simple linear function. Thus, the *z*^(1)^, *z*^(2)^ and *z*^(3)^ are the weighted sum of inputs from the input layer and the first and second hidden layers, respectively. ANNs can be made more flexible by increasing the number of hidden layers and/or the number of nodes in these layers.

### 2.3. BayCANN Algorithm

We implement BayCANN with TensorFlow to fit the ANN and Stan to obtain the parameter's posterior distributions. We use the package keras in R to create ANN metamodels that approximate the relationship between our model's input parameters and outputs and estimate the coefficients *b* and *W* (R Core Team, 2018; Jalal et al., 2017). We built the ANN from a set of probabilistic samples using a Latin hypercube sampling (LHS) design of experiment (DoE) to efficiently sample the input parameter space. Once we obtain the ANN coefficients, we perform the Bayesian calibration using the ANN rather than the simulation model.

We implemented the deep ANN in Stan (Carpenter et al., 2017) which uses a guided MCMC using gradient descent, referred to as Hamiltonian Monte-Carlo. Similarly, the R package rstan.

Both TensorFlow and Stan utilize multithreading; thus, it is essential to ensure sufficient memory is available for all threads to run efficiently.

Below we outline the steps to conduct BayCANN.

1. Structure the simulation model such that it produces outputs corresponding to the calibration targets. For example, if calibration targets are disease incidence or prevalence, ensure the model generates these outputs.
2. Generate two datasets of input parameter sets—one for training the ANN (training dataset) and the second for validating it (validation dataset). The analyst could use an LHS to efficiently sample the model inputs' prior distributions.
3. Run the simulation model using both training and validation datasets to generate their corresponding simulation model outputs.
4. Train an ANN using the training dataset, and validate it using the validation dataset. Obtaining a high-fidelity ANN is crucial to ensure getting accurate and reliable results from BayCANN (Degeling et al., 2020). Adjust the ANN's structure to obtain an accurate metamodel before proceeding.
5. Perform the Bayesian calibration by passing the ANN coefficients *W* and *b*, the prior input parameter samples, and the calibration targets to the programmed ANN framework in Stan. Stan then returns the joint posterior distribution of the calibrated parameters.

The code for implementing BayCANN is available on GitHub at https://github.com/hjalal/BayCANN. In the case study below, we use BayCANN to calibrate a colorectal cancer natural history model.

### 2.4. Case Study: Natural History Model of Colorectal Cancer

**Figure 2:**
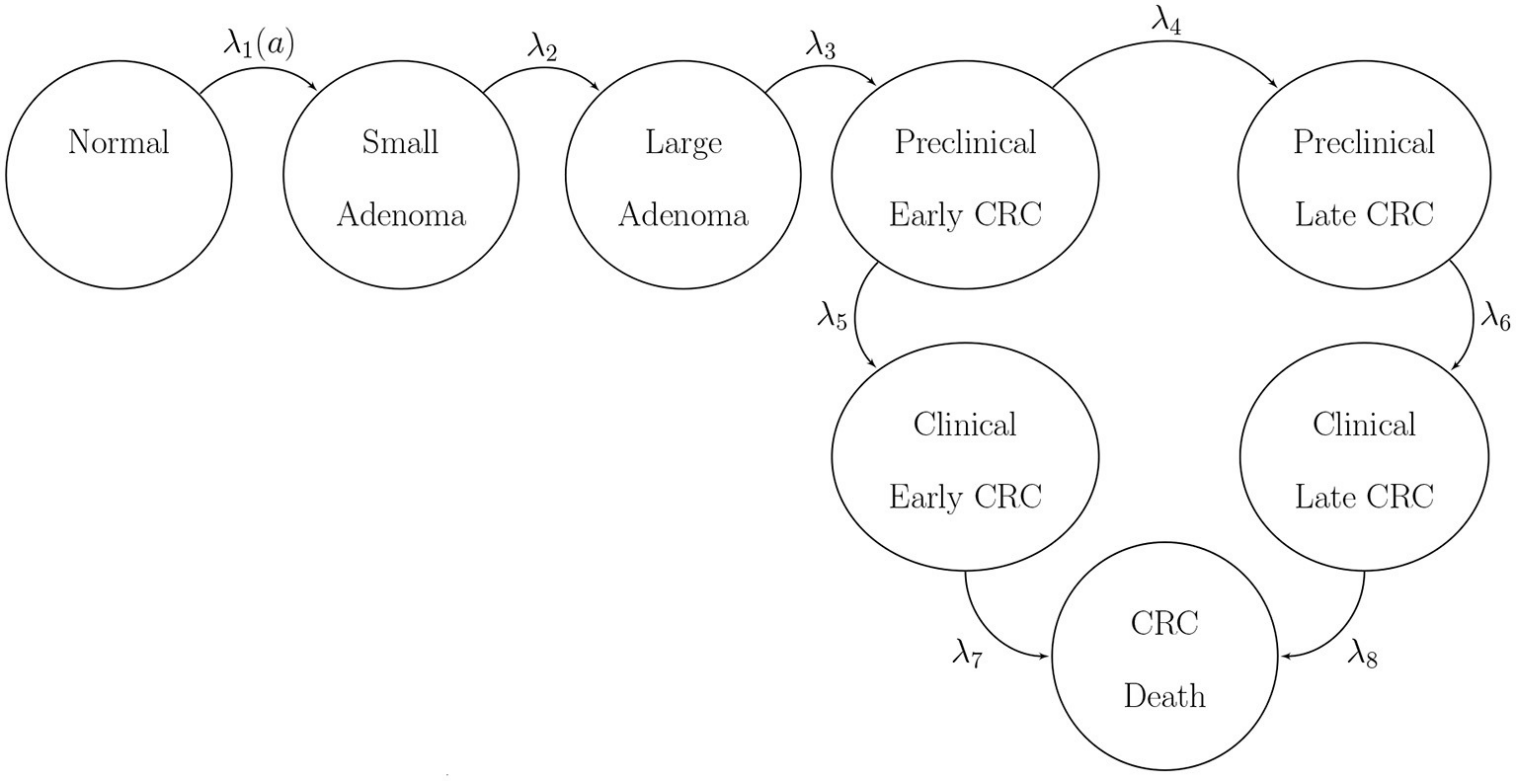
State-transition diagram of the natural history model of colorectal cancer. Ovals represent health states and arrows represent transitions. All states can transition to death from causes other than CRC with rate μ(*a*). CRC, colorectal cancer.

We use BayCANN to calibrate a state-transition model (STM) of the natural history of colorectal cancer (CRC) implemented in R (Jalal et al., 2017). We refer to our model as CRCModR. CRCModR is a discrete-time STM based on a model structure originally proposed by (Wu et al., 2006) that has previously been used for testing other methods (Alarid-Escudero et al., 2018; Heath et al., 2020). Briefly, CRCModR has 9 different health states that include absence of the disease, small and large precancerous lesions (i.e., adenomatous polyps), and early and late preclinical and clinical cancer states by stage. Figure 2 shows the state-transition diagram of the model. The progression between health states follows a continuous-time age-dependent Markov process. There are two age-dependent transition intensities (i.e., transition rates), λ_1_(*a*) and μ(*a*), that govern the age of onset of adenomas and all-cause mortality, respectively. Following Wu's original specification (Wu et al., 2006), we specify λ_1_(*a*) as a Weibull hazard such that

(5) $$\begin{array}{r} \lambda_{1}\left(a\right)=l\gamma a^{\gamma -1}, \end{array}$$

where *l* and γ are the scale and shape parameters of the Weibull hazard model, respectively. The model simulates two adenoma categories: small (adenoma smaller than or equal to 1 cm in size) and large (an adenoma larger than 1 cm in size). All adenomas start small and can transition to the large size category at a constant annual rate λ_2_. Large adenomas may become preclinical CRC at a constant annual rate λ_3_. Both small and large adenomas may progress to preclinical CRC, although most will not in an individual's lifetime. Early preclinical cancers progress to late stages at a constant annual rate λ_4_ and could become symptomatic at a constant annual rate λ_5_. Late preclinical cancer could become symptomatic at a constant annual rate λ_6_. After clinical detection, the model simulates the survival time to death from early and late CRC using time-homogeneous mortality rates, λ_7_ and λ_8_, respectively. In total, the model has nine health states: normal, small adenoma, large adenoma, early preclinical CRC, late preclinical CRC, CRC death, and other causes of death. The state-transition diagram of the model is shown in Figure 2. The model simulates the natural history of CRC of a hypothetical cohort of 50-year-old women in the U.S. over a lifetime. The cohort starts the simulation with a prevalence of adenoma of *p*_*adeno*_. A proportion, *p*_*small*_, corresponds to small adenomas and prevalence of preclinical early and late CRC of 0.12 and 0.08, respectively. The simulated cohort in any state is at risk of all-cause mortality μ(*a*) obtained from the U.S. life tables Arias (2014). Similar models to CRCmodR have been used to inform population-level screening guidelines in the U.S. (Knudsen et al., 2016).

CRCModR has 11 parameters summarized in Table 2 (Alarid-Escudero et al., 2018). Mortality rates from early and late stages of CRC (λ_7_, λ_8_]) could be obtained from cancer population registries (e.g., SEER in the U.S.). Thus, we calibrate the model to the remaining nine parameters (*p*_*adeno*_, *p*_*small*_, *l*,γ, λ_2_, λ_3_, λ_4_, λ_5_ and λ_6_).

**Table 2:** The parameters of the natural history model of colorectal cancer (CRC).

**Parameter**	**Description**	**Base value**	**Calibrate?**	**Source**	**Prior range**
*l*	Scale parameter of Weibull hazard	2.86e-06	Yes	Wu et al., 2006	[2 × 10^−6^, 2 × 10^−5^]
*g*	Shape parameter of Weibull hazard	2.78	Yes	Wu et al., 2006	[2.00, 4.00]
λ_2_	Small adenoma to large adenoma	0.0346	Yes	Wu et al., 2006	[0.01, 0.10]
λ_3_	Large adenoma to preclinical early CRC	0.0215	Yes	Wu et al., 2006	[0.01, 0.04]
λ_4_	Preclinical early to preclinical late CRC	0.3697	Yes	Wu et al., 2006	[0.20, 0.50]
λ_5_	Preclinical early to clinical early CRC	0.2382	Yes	Wu et al., 2006	[0.20, 0.30]
λ_6_	Preclinical late to clinical late CRC	0.4852	Yes	Wu et al., 2006	[0.30, 0.70]
λ_7_	CRC mortality in early stage	0.0302	No	Wu et al., 2006	-
λ_8_	CRC mortality in late stage	0.2099	No	Wu et al., 2006	-
*p*_*adeno*_	Prevalence of adenoma at age 50	0.27	Yes	Rutter et al., 2007	[0.25, 0.35]
*p*_*small*_	Proportion of small adenomas at age 50	0.71	Yes	Wu et al., 2006	[0.38, 0.95]

*The base values are used to generate the calibration targets and the ranges of the uniform distribution used as priors for the Bayesian calibration*.

#### 2.4.1. Confirmatory Analysis

**Figure 3:**
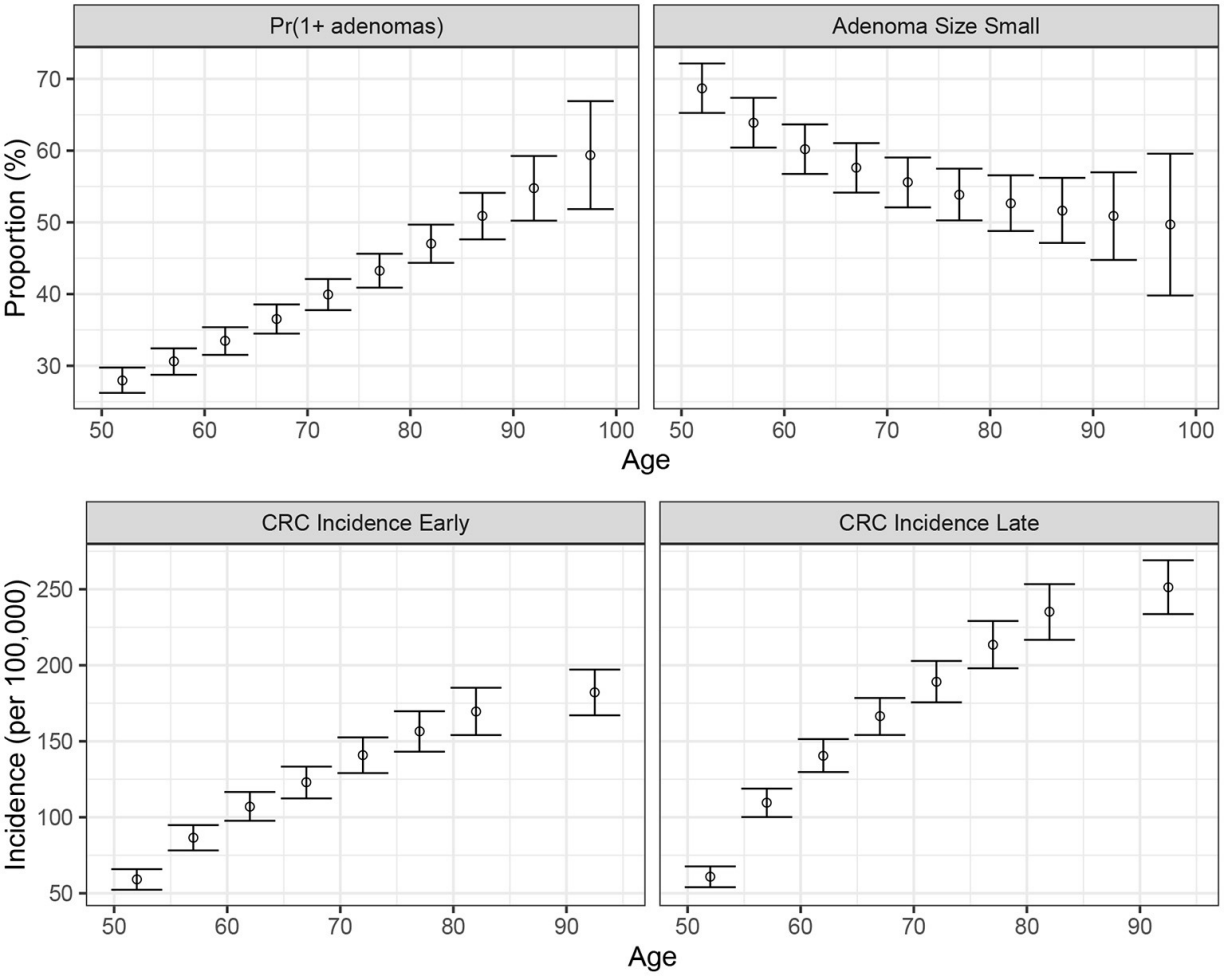
Generated calibration targets and its 95% credible interval of a cohort of 500 and 100,000 simulated individuals for adenoma-related targets cancer incidence targets, respectively plotted against age in years on the x-axis. These distributions are from 100 different runs using the same parameter set values in each set of runs.

We conducted a confirmatory analysis to compare BayCANN vs. IMIS. To obtain the “truth” that we could compare BayCANN and IMIS against, we *generated* the synthetic targets using the base-case values in Table 2. We generated four age-specific targets, including adenoma prevalence, the proportion of small adenomas, and CRC incidence for early and late stages which represent commonly used calibration targets for this type of model (Kuntz et al., 2011). To generate the calibration targets, we ran CRCModR as a microsimulation (Krijkamp et al., 2018) 100 times to produce different adenoma-related and cancer incidence outputs. We then aggregated the results across all 100 outputs to compute their mean and standard errors (SE). Different calibration targets could have different levels of uncertainty given the amount of data to compute their summary measures. Therefore, to account for different variations in the amount of data on different calibration targets, we simulated different numbers of individuals for adenoma targets (*N* = 500) and cancer incidence targets (*N* = 100, 000). Figure 3 shows the generated adenoma-related and cancer incidence calibration targets aggregated over 100 different runs using the parameter set in Table 2.

To create a deep ANN metamodel, we generated a DOE by sampling each of the nine parameters from the ranges of the uniform distributions using an LHS design as shown in Table 2. Specifically, we created two LHS input datasets of sizes 8,000 samples and 2,000 samples for training and validating the ANN, respectively. We then ran the natural history model and generated adenoma prevalence and CRC incidence for each parameter set.

We define an ANN with two hidden layers and 100 nodes per hidden layer. Then, we evaluated the ANN's performance by validating the predicted values for the 36 outcomes against the observed values from the validation dataset. The likelihood function for BayCANN was constructed by assuming that the targets, *y*_*t*_*i*__, are normally distributed with mean ϕ_*t*_*i*__ and standard deviation σ_*t*_*i*__, where ϕ_*t*_*i*__ = *M*[θ] is the model-predicted output for each type of target *t* and age group *i* at parameter set θ. We defined uniform prior distributions for all θ_*u*_ based on previous knowledge or nature of the parameters (Table 2).

We compare BayCANN against a full Bayesian calibration of the natural history model using the incremental mixture importance sampling (IMIS) algorithm. The IMIS algorithm has been described elsewhere (Raftery and Bao, 2010), but briefly, this algorithm reduces the computational burden of Bayesian calibration by incrementally building a better importance sampling function based on Gaussian mixtures.

## 3. Results

We present the ANN's performance in approximating the output of the simulation model and compare the generated joint posterior distribution of the simulation model parameters produced from BayCANN against the full joint posterior from the IMIS approach. We compare both BayCANN and IMIS results recovering the “true” values—the parameter values we used to generate the calibration targets in the confirmatory analysis.

### 3.1. Validation

**Figure 4:**
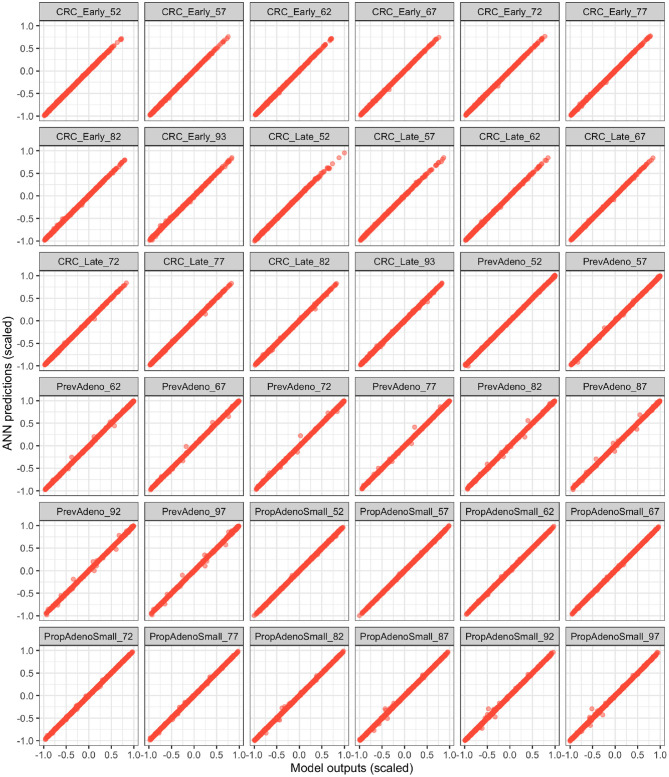
Validation of the fitted ANN on the validation Latin hyper cube sample (LHS) dataset. The x and y axes represent the scaled model outputs and scaled ANN predictions, respectively.

Figure 4 illustrates the ANN's performance in predicting the model outputs using the validation dataset. Each plot represents one of the model outputs, where we compare the ANN's prediction on the y-axis against the model's output on the x-axis. Each red dot represents one of the 2,000 DOE validation samples not used to train the ANN. The ANN had a high prediction performance in approximating the model outputs (*R*^2^ > 99.9%), indicating that the deep ANN is a high fidelity metamodel of the simulation model within the parameter ranges we evaluated.

### 3.2. Comparing BayCANN and IMIS

**Figure 5:**
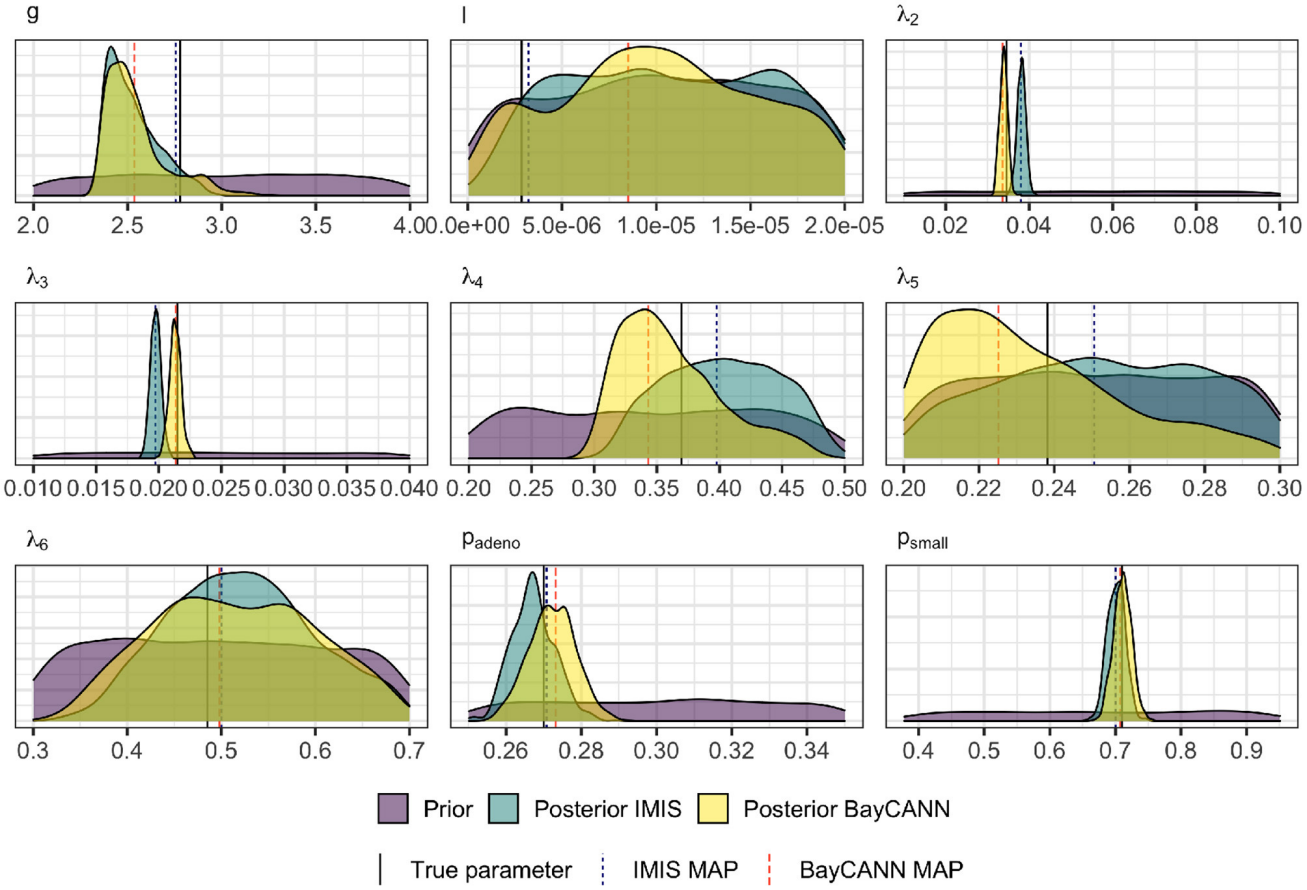
Prior and Marginal posterior distributions of the calibrated parameters from the IMIS and BayCANN methods. The vertical solid lines indicate the “true” parameter values (i.e., the value of the parameters used to generate the calibration targets in the confirmatory analysis). The vertical dashed lines represent the maximum a posteriori (MAP) for BayCANN and the incremental mixture importance sampling (IMIS).

Figure 5 compares BayCANN against IMIS in recovering the true parameter values used to generate the targets. The 95% credible intervals (CrI) of each parameter distribution obtained from BayCANN cover all nine true parameters. For IMIS, the 95% CrI did not cover the true parameters for λ_2_ and λ_3_. This figure also shows the maximum a posteriori (MAP) estimate for both BayCANN and IMIS. The MAP is the sample associated with the highest log-posterior and indicates the posterior parameter set that best fits the target data.

**Figure 6:**
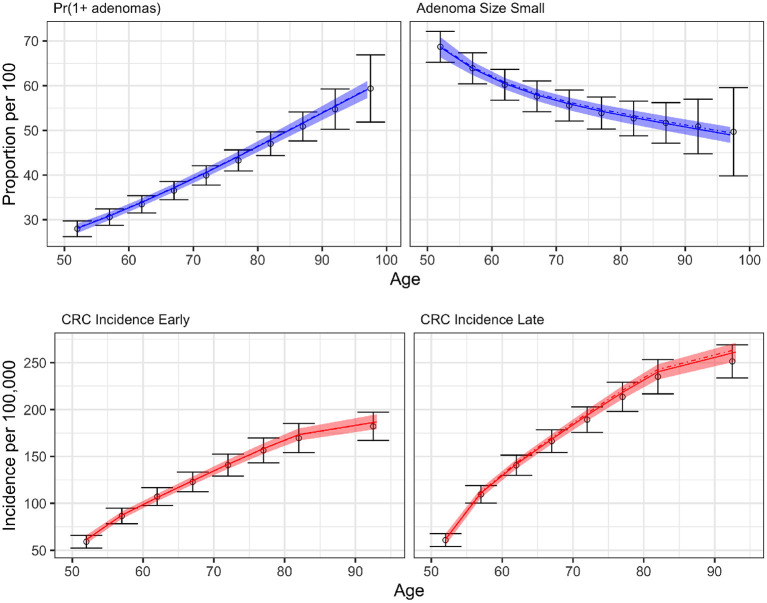
BayCANN calibration results by age in years on the x-axis. The upper panels show adenoma targets and lower panels show cancer incidence targets by stage. Calibration targets with their 95% confidence intervals are shown in black. The colored curves show the posterior model-predicted mean, and the shaded area shows the corresponding 95% posterior model-predicted credible interval of the outcomes. The dashed-dotted lines represent the output using the maximum a posteriori (MAP) estimate from BayCANN.

Figure 6 compares the results of BayCANN against all the calibration targets for the probability of developing multiple adenomas, the proportion of small adenomas, and early and late clinical CRC incidence. Upon visual inspection, BayCANN fits all calibration targets well, indicating that the joint posterior distribution from BayCANN can produce targets in the desired ranges. The results here represent the model-predictive mean and the credible interval of using 10,000 posterior samples from BayCANN. We also present the results of using BayCANN's MAP estimates which closely follow the model-predicted posterior mean from the 10,000 posterior samples.

In this example, BayCANN was five times faster than the IMIS. The IMIS algorithm took 80 min to run in a MacBook Pro Retina, 15-inch, Late 2013 with a 2.6 GHz Intel Core i7 processor with 4 cores and 16 gigabytes of RAM. BayCANN took only 15 min on the same computer; 5 min to produce 10,000 samples for both LHS DOE dataset generations and about 10 min to fit the ANN in TensorFlow and produce the joint posterior distributions in Stan. The computational gain of BayCANN was modest given that our case study model was efficient and deterministic.

**Figure 7:**
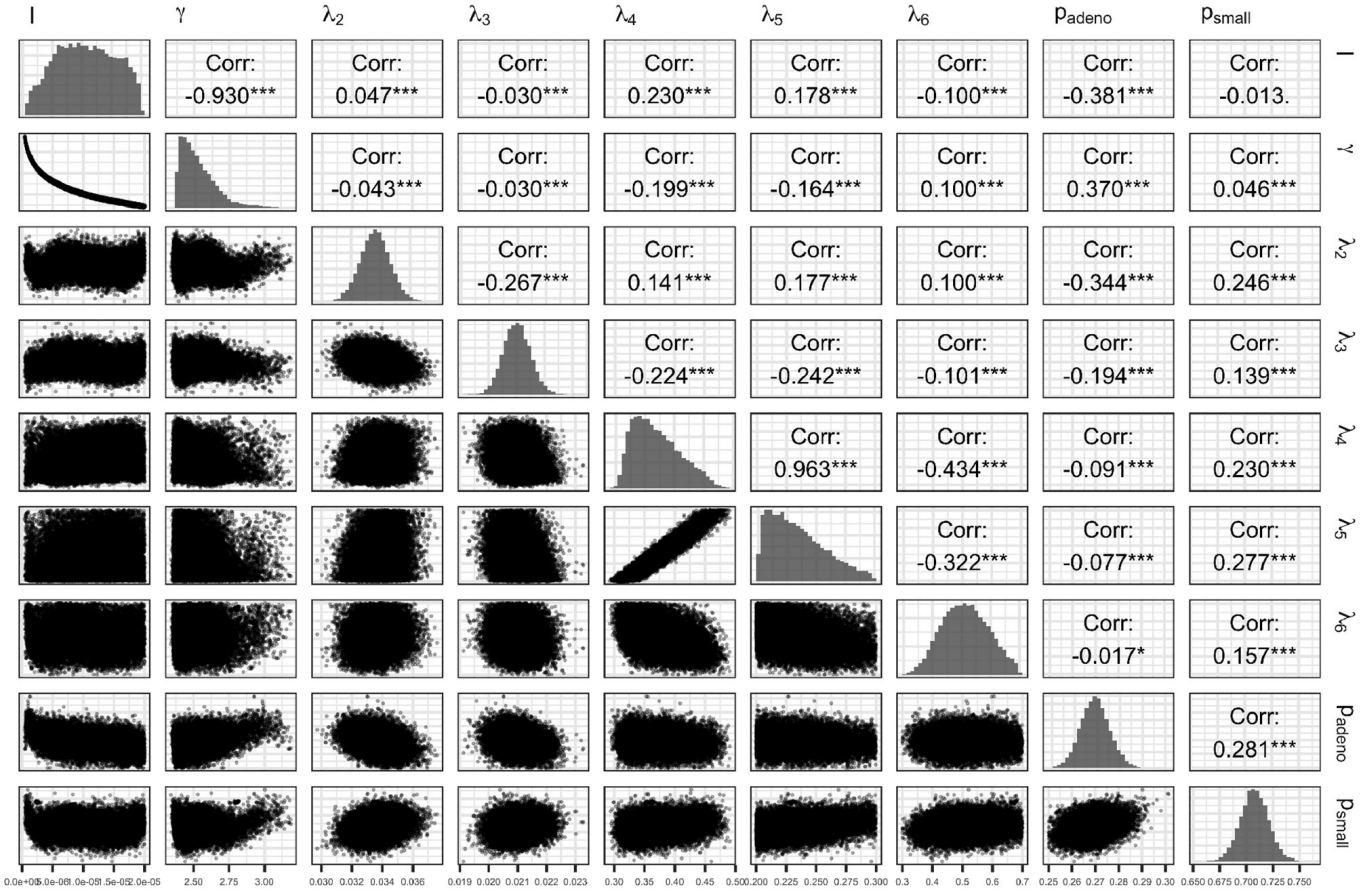
Joint posterior distribution of the calibrated parameters of the case study using the ANN method.

Figure 7 presents the joint distribution of all pairwise parameters in BayCANN, and along the diagonal, the marginal distributions of each parameter. This figure reveals insightful information about this calibration exercise. In practice, many calibrated parameters are correlated as shown in this figure. The absolute value of these correlations range from 0.013 to 0.963. The strength of the correlation reflects the level of non-identifiability between that pair of parameters. The stronger the correlation the higher the non-identifiability and the greater need to add additional target data or modify the model structure to *separate* the parameters in question (Alarid-Escudero et al., 2018).

## 4. Discussion

In this study, we propose BayCANN as a feasible and practical solution to Bayesian calibration challenges in complex health decision science models. The distinct advantage of using BayCANN is that it represents the model on a functional basis as an ANN. Then, the ANN can become a high-fidelity representation of the model. Thus, those interested in implementing BayCANN can do so without the need to code their models in a probabilistic programming language. Given the high computational efficiency of the ANN, BayCANN can also provide a computational advantages over other Bayesian calibration methods.

BayCANN uses ANNs specifically to streamline Bayesian calibration. ANNs have also been used as metamodels of both stochastic and deterministic responses, mainly for their computational efficiency (Barton, 2009; Badiru and Sieger, 1998; Hurrion, 1997; Chambers and Mount-Campbell, 2002; Zobel and Keeling, 2008). One of the first implementations of ANN as metamodels was in 1992 for a scheduling simulation model (Pierreval et al., 1992; Pierreval and Huntsinger, 1992). Since then, ANNs have been successfully implemented as emulators of all sorts of discrete-event and continuous simulation models in a wide variety of fields (Kilmer, 1996; Sabuncuoglu and Touhami, 2002; Fonseca et al., 2003; El Tabach et al., 2007). ANNs have also been proposed as proxies for non-linear and simulation models (Paiva et al., 2010; Mareš and Kučerová, 2012; Pichler et al., 2003). An example of ANNs as metamodels is estimating the mean and variance of patient time in emergency department visits (Kilmer, 1994; Kilmer et al., 1997). Nowadays, ANNs are widely popular as machine learning tools in artificial intelligence (Schmidhuber, 2015). Deep learning using ANNs are used for visual recognition in self-driving cars (Ndikumana et al., 2020) and in classifying galaxies (Folkes et al., 1996). ANNs have been used for calibration of computationally expensive models, such as general circulation and rainfall-runoff models in climate science (Khu et al., 2004; Hauser et al., 2012), and other complex global optimization techniques such as genetic algorithms (Wang, 2005).

The superior performance of BayCANN relative to IMIS may pertain to the bias of the ANN in BayCANN being relatively lower than that of the Bayesian approximation of IMIS. BayCANN uses ANNs as high-fidelity metamodels of the simulator and conducts full Bayesian calibration. However, IMIS is an approximation of Bayesian inference that directly uses the simulator itself. Thus, visual examination of the ANN's performance similar to Figure 4 is an important step to ensure obtaining high-fidelity ANN for BayCANN.

Bayesian calibration provides other practical advantages over direct-search algorithms because the samples from the joint posterior distribution can be used directly as inputs to probabilistic sensitivity analyses (PSA) which are now required for cost-effectiveness analyses (Neumann et al., 2016; Rutter et al., 2019). This joint posterior distribution is also informative in non-identifiable calibration problems where calibration targets are not sufficient to provide a unique solution to the calibrated parameters. Non-identifiability is often overlooked using standard non-Bayesian calibration approaches (Alarid-Escudero et al., 2018).

In our case study, BayCANN was both faster and overall more accurate in recovering the true parameter values than the IMIS algorithm. We developed BayCANN to be generalizable to models of various complexities, and we provide the open-source implementation in R and Stan to facilitate its wider adoption.

BayCANN may have an additional advantage for representing models with first-order Monte-Carlo noise from individual-based state-transition models (iSTM). Traditionally, calibrating these models has been challenging because of (1) the stochasticity of each simulation due to the simulator's output varying given the same set of input parameter values, and (2) the extra computational burden involved in calibrating iSTM. Because BayCANN averages over a set of simulations, it can account for the first-order Monte-Carlo noise. Further research is needed to study BayCANN's performance in stochastic models.

We chose ANNs over other metamodeling techniques because of their flexibility, efficiency, and ability to accept a large number of inputs. The use of metamodels in Bayesian calibration has been mostly limited to Gaussian processes (GP) (Kennedy and O'Hagan, 2001; Gramacy, 2020). GPs are attractive because they can be specified fully as Bayesian models (Kennedy and O'Hagan, 2001). However, GPs are not without limitations, the main one being that they are themselves relatively computationally expensive. In practice, computational challenges limit training GPs to datasets in the low thousands limiting their applicability to health decision sciences models (Gramacy, 2020).

Our approach has some limitations. First, ANNs are not fully probabilistic, thus, the joint posterior distribution produced from the Bayesian calibration is an approximation of the true distribution. Other metamodels, such as GPs are fully probabilistic and can produce the full joint posterior distribution (Gramacy, 2020). However, applying GPs in complex models can be computationally infeasible (Gramacy, 2020). Second, accuracy—Because ANNs (and GPs) are metamodels, they may rarely achieve 100% precision compared to using the simulation model itself. In our example, with a relatively simple ANN (only two hidden layers with 100 hidden nodes each), we were able to achieve 99.9% accuracy. However, for other application, the accuracy of the ANN might be lower especially if the model outputs are not continuous or smooth in certain region of the parameter space. In addition, over-fitting can be a serious problem with any metamodel especially when the purpose of the metamodel is as sensitive as calibration. To reduce the chance of over-fitting, we validated the model against a subset of simulation runs. We visually inspected the degree of fit for the simulation output against those predicted by the ANN (Figure 4). Third, similar to any Bayesian model, the choice of priors can be important. Fortunately, in health decision sciences' models, analysts often make careful choices of their priors when designing their models and running PSA analyses. Additionally, the best-fitting parameters may be outside the simulated ranges. Notably, the joint posterior distribution can give insights into the parameter ranges. For example, if a parameter is skewed heavily without a clear peak, that may indicate that the parameter range needs to be shifted to cover values that may fit better. This process is usually iterative and may involve multiple steps or redefining the parameter ranges and recalibrating the model. Finally, there is no strict guideline for choosing the number of hidden ANN layers or the number of nodes per layer. In this study, we chose an ANN with two hidden layers and 100 nodes per layer. Adjusting these parameters and additional parameters of the Bayesian calibration process can improve the calibration results and can be easily changed in BayCANN. While determining these values apriori can be challenging, we recommend modelers who wish to use BayCANN to start with simple settings and gradually increase the complexity of the ANN to accommodate their particular needs. We provide flexible code in R and Stan to simplify these tasks.

In summary, Bayesian calibration can reveal important insights into model parameter values and produce outcomes that match observed data. BayCANN is one effort to target the computational and technical challenges of Bayesian calibration for complex models.

## Data Availability Statement

The original contributions presented in the study are included in the article and the code for BayCANN is available at https://github.com/hjalal/BayCANN, further inquiries can be directed to the corresponding author.

## Author Contributions

HJ and FA-E conceived the study. HJ, FA-E, and TT conducted the analyses, contributed to interpreting the results, and writing the manuscript. All authors contributed to the article and approved the submitted version.

## Conflict of Interest

The authors declare that the research was conducted in the absence of any commercial or financial relationships that could be construed as a potential conflict of interest.
